# Crystal structure of 2-cyano-*N*-(furan-2-ylmeth­yl)-3-(3-nitro­phen­yl)propanamide

**DOI:** 10.1107/S2056989015012918

**Published:** 2015-07-15

**Authors:** Shivanna Subhadramma, Budanur Papaiah Siddaraju, Naveen Chandra, Janardhanan Saravanan, Dasararaju Gayathri

**Affiliations:** a Department of Physics, Dr M. G. R. Educational and Research Institute University, Maduravoyal, Chennai, India; b Department of Engineering Chemistry, Cauvery Institute of Technology, Sundhahalli, Mandya, India; c Department of Chemistry, Post-Graduate and Research Centre, St Joseph’s College (Autonomous), Bangalore 560 027, India; d Department of Pharmaceutical Chemistry, PES College of Pharmacy, Hanumanthnagar, Bangalore 560 050, India; e Centre of Advanced Study in Crystallography and Biophysics, University of Madras, Guindy Campus, Chennai 600 025, India

**Keywords:** crystal structure, furan, acetamide, N—H⋯N hydrogen bonds, inversion dimers

## Abstract

In the title compound, C_15_H_11_N_3_O_4_, the acetamide group is inclined to the furan ring by 66.5 (1)°. The dihedral angle between the furan ring and the benzene ring is 66.8 (1)°. In the crystal, mol­ecules are linked by pairs of N—H⋯N hydrogen bonds, forming inversion dimers with an *R*_2_^2^(12) ring motif. The dimers are linked *via* two pairs of C—H⋯O hydrogen bonds to the same acceptor oxygen atom, enclosing *R*_2_^1^(6) ring motifs, forming chains along the [101] direction.

## Related literature   

For examples of biological properties of furan derivatives, see: Anupam *et al.* (2011 ▸). For the biological activities of some heterocyclic derivatives containing the acetamide moiety, see: Fallah-Tafti *et al.* (2011 ▸); Shams *et al.* (2011 ▸). For the crystal structure of the similar compound 2-cyano-*N*-furfuryl-3-(2-fur­yl)acryl­amide, see: Pomés Hernández *et al.* (1996 ▸).
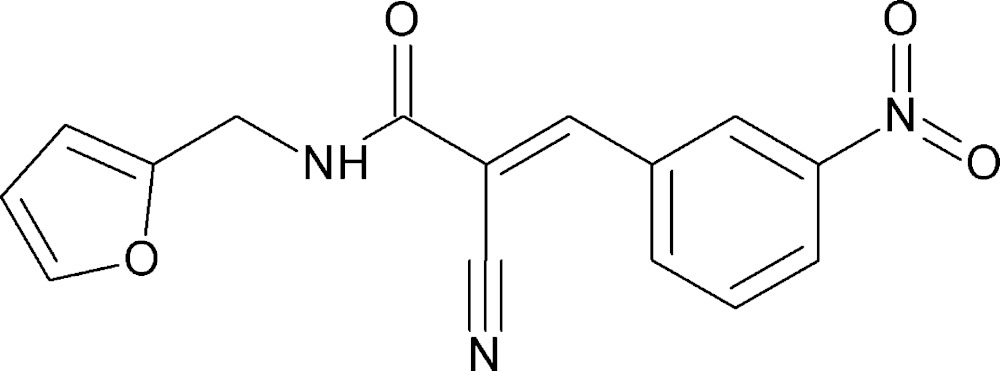


## Experimental   

### Crystal data  

C_15_H_11_N_3_O_4_
*M* *_r_* = 297.27
Triclinic, 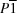

*a* = 7.4358 (3) Å
*b* = 9.4165 (5) Å
*c* = 10.3934 (5) Å
α = 90.938 (2)°
β = 96.910 (2)°
γ = 105.872 (2)°
*V* = 693.98 (6) Å^3^
*Z* = 2
Mo *K*α radiation
μ = 0.11 mm^−1^
*T* = 293 K
0.30 × 0.20 × 0.20 mm

### Data collection  

Bruker Kappa APEXII CCD diffractometer
Absorption correction: multi-scan (*SADABS*; Bruker, 2004 ▸) *T*_min_ = 0.942, *T*_max_ = 0.983
12708 measured reflections
2442 independent reflections
2055 reflections with *I* > 2σ(*I*)
*R*_int_ = 0.027

### Refinement  

*R*[*F* ^2^ > 2σ(*F* ^2^)] = 0.039
*wR*(*F* ^2^) = 0.116
*S* = 1.02
2442 reflections
200 parameters
H-atom parameters constrained
Δρ_max_ = 0.22 e Å^−3^
Δρ_min_ = −0.16 e Å^−3^

### 

Data collection: *APEX2* (Bruker, 2004 ▸); cell refinement: *APEX2* and *SAINT* (Bruker, 2004 ▸); data reduction: *SAINT* and *XPREP* (Bruker, 2004 ▸); program(s) used to solve structure: *SHELXS2014* (Sheldrick, 2008 ▸); program(s) used to refine structure: *SHELXL2014* (Sheldrick, 2015 ▸); molecular graphics: *PLATON* (Spek, 2009 ▸); software used to prepare material for publication: *SHELXL2014* and *PLATON*.

## Supplementary Material

Crystal structure: contains datablock(s) I, global. DOI: 10.1107/S2056989015012918/su5161sup1.cif

Structure factors: contains datablock(s) I. DOI: 10.1107/S2056989015012918/su5161Isup2.hkl

Supporting information file. DOI: 10.1107/S2056989015012918/su5161Isup3.cml

**Fig. 1.**
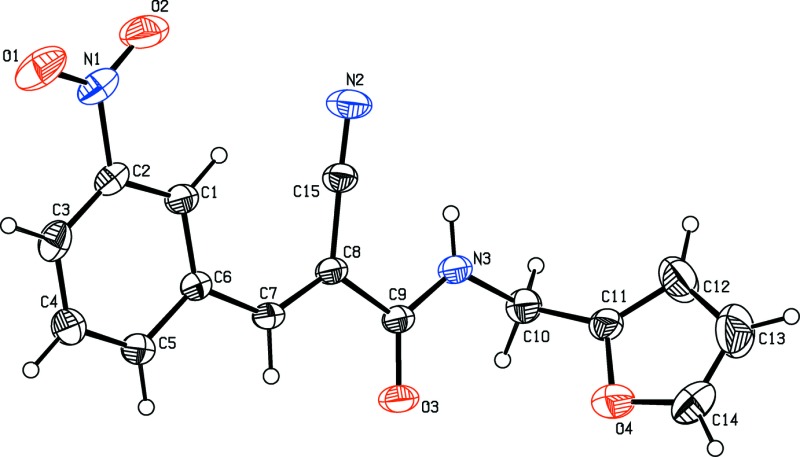
The molecular structure of the title compound, with atom labelling. Displacement ellipsoids are drawn at the 30% probability level.

**Fig. 2.**
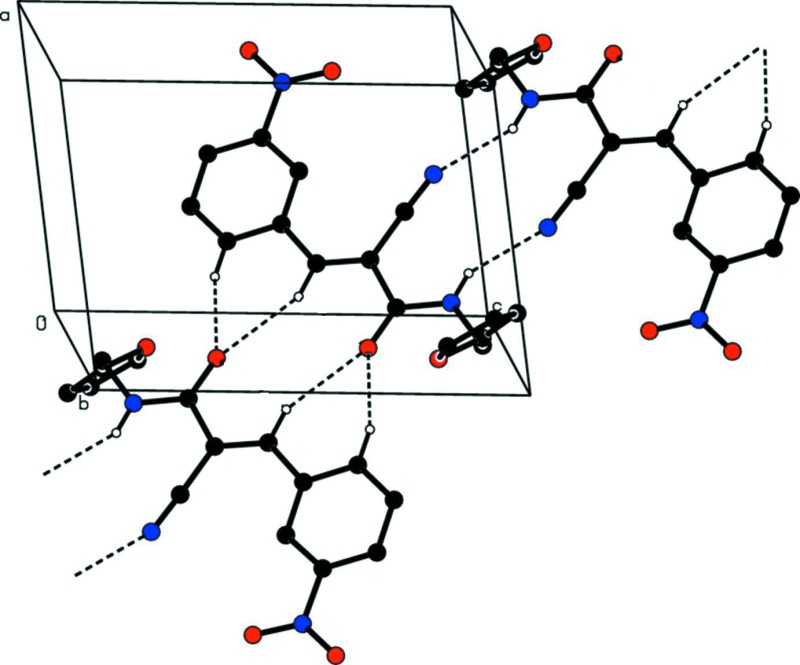
A view along the b axis of the crystal packing of the title compound. Hydrogen bonds are shown as dashed lines (see Table 1 for details). For clarity, H atoms not involved in hydrogen bonding have been omitted.

. DOI: 10.1107/S2056989015012918/su5161fig1.tif
The mol­ecular structure of the title compound, with atom labelling. Displacement ellipsoids are drawn at the 30% probability level.

b . DOI: 10.1107/S2056989015012918/su5161fig2.tif
A view along the *b* axis of the crystal packing of the title compound. Hydrogen bonds are shown as dashed lines (see Table 1 for details). For clarity, H atoms not involved in hydrogen bonding have been omitted.

CCDC reference: 1410650

Additional supporting information:  crystallographic information; 3D view; checkCIF report

## Figures and Tables

**Table 1:** Hydrogen-bond geometry (, )

*D*H*A*	*D*H	H*A*	*D* *A*	*D*H*A*
N3H3*A*N2^i^	0.86	2.27	3.056(2)	152
C5H5O3^ii^	0.93	2.49	3.337(2)	151
C7H7O3^ii^	0.93	2.49	3.362(2)	156

Symmetry codes: (i) 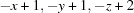; (ii) 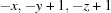.

## supplementary crystallographic information

### S1. Comment

Furan is one of the most important five-membered heterocyclic ring systems and its derivatives are well known to possess various biological properties, such as antibacterial, antitumor, anti-inflammatory, antifungal, anticonvulsant, and analgesic (Anupam *et al.*, 2011). Acetamide derivatives possess a wide range of pharmacological properties (Fallah-Tafti *et al.*, 2011; Shams *et al.*, 2011). In view of the biological importance of furan and acetamide derivatives, we have synthesized the title compound and report herein on its crystal structure.

In the title compound, Fig. 1, the acetamide group is inclined to the furan ring by 66.5 (1)°. Torsion angles N3—C9—C8—C15 [-5.4 (2)°] and O3—C9—C8—C15 [174.6 (2)°] indicate that the acetonitrile and acetamide moieties are almost planar. The dihedral angle between the furan ring and the benzene ring is 66.8 (1)°. The bond lengths and bond angles are comparable with those reported for a similar structure, *viz.* 2-cyano-*N*-furfuryl-3-(2-furyl)acrylamide (Pomés Hernández *et al.*, 1996).

In the crystal (Table 1 and Fig. 2), molecules are linked by pairs of N-H···N hydrogen bonds forming inversion dimers with an R_2_^2^(12) ring motif. The dimers are linked *via* two pairs of bifurcated C-H···O hydrogen bonds, enclosing *R*_2_^1^(6) ring motifs, forming chains along direction [101].

### S2. Experimental

An equimolar mixture of furfuryl amine and ethyl cyano acetate was mixed in a conical flask and the mixture was heated under microwave irradiation at 700 W for 3 min with an interval of 20 s each time. The mixture was then poured into a beaker and cooled giving a solid that was washed with ethanol. The furfuryl cyano acetamide product so obtained was treated with an equi-molar ratio of 3-nitro benzaldehyde in the presence of glacial acetic acid and refluxed for 3 h. On cooling, colourless crystals of the title compound were obtained.

### S3. Refinement

Crystal data, data collection and structure refinement details are summarized in Table 2. The NH and C-bound H atoms were included in calculated positions and refined using a riding model: N—H = 0.86 Å, C—H = 0.93 - 0.97 Å with *U*_iso_(H) = 1.2*U*_eq_(N,C).

### Crystal data

**Table:**

C_15_H_11_N_3_O_4_	*Z* = 2
*M**_r_* = 297.27	*F*(000) = 308
Triclinic, *P*1	*D*_x_ = 1.423 Mg m^−^^3^
*a* = 7.4358 (3) Å	Mo *K*α radiation, λ = 0.71073 Å
*b* = 9.4165 (5) Å	Cell parameters from 6452 reflections
*c* = 10.3934 (5) Å	θ = 2.8–25.6°
α = 90.938 (2)°	µ = 0.11 mm^−^^1^
β = 96.910 (2)°	*T* = 293 K
γ = 105.872 (2)°	Block, colourless
*V* = 693.98 (6) Å^3^	0.30 × 0.20 × 0.20 mm

### Data collection

**Table:**

Bruker Kappa APEXII CCD diffractometer	2442 independent reflections
Radiation source: fine-focus sealed tube	2055 reflections with *I* > 2σ(*I*)
Graphite monochromator	*R*_int_ = 0.027
ω and φ scan	θ_max_ = 25.0°, θ_min_ = 2.0°
Absorption correction: multi-scan (*SADABS*; Bruker, 2004)	*h* = −8→8
*T*_min_ = 0.942, *T*_max_ = 0.983	*k* = −11→10
12708 measured reflections	*l* = −12→12

### Refinement

**Table:**

Refinement on *F*^2^	Secondary atom site location: difference Fourier map
Least-squares matrix: full	Hydrogen site location: inferred from neighbouring sites
*R*[*F*^2^ > 2σ(*F*^2^)] = 0.039	H-atom parameters constrained
*wR*(*F*^2^) = 0.116	*w* = 1/[σ^2^(*F*_o_^2^) + (0.059*P*)^2^ + 0.2005*P*] where *P* = (*F*_o_^2^ + 2*F*_c_^2^)/3
*S* = 1.02	(Δ/σ)_max_ < 0.001
2442 reflections	Δρ_max_ = 0.22 e Å^−^^3^
200 parameters	Δρ_min_ = −0.16 e Å^−^^3^
0 restraints	Extinction correction: *SHELXL2014* (Sheldrick, 2015), Fc^*^=kFc[1+0.001xFc^2^λ^3^/sin(2θ)]^-1/4^
Primary atom site location: structure-invariant direct methods	Extinction coefficient: 0.030 (5)

### Special details

**Table:**

Geometry. All e.s.d.'s (except the e.s.d. in the dihedral angle between two l.s. planes) are estimated using the full covariance matrix. The cell e.s.d.'s are taken into account individually in the estimation of e.s.d.'s in distances, angles and torsion angles; correlations between e.s.d.'s in cell parameters are only used when they are defined by crystal symmetry. An approximate (isotropic) treatment of cell e.s.d.'s is used for estimating e.s.d.'s involving l.s. planes.

### Fractional atomic coordinates and isotropic or equivalent isotropic displacement parameters (Å^2^)

**Table:**

	*x*	*y*	*z*	*U*_iso_*/*U*_eq_	
C1	0.5353 (2)	0.28891 (17)	0.58091 (15)	0.0437 (4)	
H1	0.5862	0.3239	0.6654	0.052*	
C2	0.6211 (2)	0.20823 (17)	0.51113 (16)	0.0450 (4)	
C3	0.5534 (3)	0.15471 (19)	0.38555 (17)	0.0548 (5)	
H3	0.6160	0.1015	0.3400	0.066*	
C4	0.3898 (3)	0.1826 (2)	0.32955 (17)	0.0576 (5)	
H4	0.3401	0.1470	0.2450	0.069*	
C5	0.2988 (2)	0.26268 (18)	0.39767 (15)	0.0479 (4)	
H5	0.1879	0.2798	0.3586	0.057*	
C6	0.3703 (2)	0.31818 (16)	0.52382 (14)	0.0389 (4)	
C7	0.2662 (2)	0.40380 (16)	0.58752 (14)	0.0392 (4)	
H7	0.1617	0.4177	0.5360	0.047*	
C8	0.2965 (2)	0.46539 (16)	0.70769 (14)	0.0380 (4)	
C9	0.1628 (2)	0.54718 (17)	0.74909 (14)	0.0404 (4)	
C10	0.0773 (2)	0.67695 (19)	0.92842 (16)	0.0486 (4)	
H10A	0.0750	0.6564	1.0194	0.058*	
H10B	−0.0509	0.6412	0.8851	0.058*	
C11	0.1429 (2)	0.83840 (19)	0.91721 (15)	0.0474 (4)	
C12	0.2518 (5)	0.9468 (3)	0.9961 (2)	0.1020 (10)	
H12	0.3096	0.9378	1.0788	0.122*	
C13	0.2647 (5)	1.0791 (3)	0.9319 (3)	0.1089 (10)	
H13	0.3310	1.1739	0.9645	0.131*	
C14	0.1663 (3)	1.0432 (3)	0.8186 (3)	0.0790 (7)	
H14	0.1519	1.1094	0.7553	0.095*	
C15	0.4516 (2)	0.45908 (19)	0.80123 (14)	0.0447 (4)	
N1	0.7935 (2)	0.17794 (17)	0.57526 (18)	0.0592 (4)	
N2	0.5728 (2)	0.4565 (2)	0.87857 (14)	0.0667 (5)	
N3	0.19322 (18)	0.59563 (15)	0.87343 (12)	0.0439 (3)	
H3A	0.2851	0.5781	0.9231	0.053*	
O1	0.8792 (2)	0.1167 (2)	0.51177 (17)	0.0881 (5)	
O2	0.8406 (2)	0.2139 (2)	0.69036 (16)	0.0840 (5)	
O3	0.03619 (18)	0.56592 (15)	0.67145 (11)	0.0609 (4)	
O4	0.0876 (2)	0.89464 (16)	0.80594 (13)	0.0724 (4)	

### Atomic displacement parameters (Å^2^)

**Table:**

	*U*^11^	*U*^22^	*U*^33^	*U*^12^	*U*^13^	*U*^23^
C1	0.0448 (9)	0.0476 (9)	0.0417 (8)	0.0195 (7)	0.0013 (7)	0.0035 (7)
C2	0.0437 (9)	0.0420 (9)	0.0556 (10)	0.0200 (7)	0.0106 (7)	0.0120 (7)
C3	0.0667 (11)	0.0488 (10)	0.0584 (11)	0.0261 (9)	0.0212 (9)	0.0027 (8)
C4	0.0688 (12)	0.0622 (11)	0.0452 (9)	0.0268 (9)	0.0017 (8)	−0.0086 (8)
C5	0.0490 (9)	0.0522 (10)	0.0439 (9)	0.0201 (8)	−0.0023 (7)	−0.0022 (7)
C6	0.0395 (8)	0.0396 (8)	0.0392 (8)	0.0148 (6)	0.0023 (6)	0.0039 (6)
C7	0.0376 (8)	0.0436 (8)	0.0381 (8)	0.0172 (7)	−0.0022 (6)	0.0032 (6)
C8	0.0375 (8)	0.0413 (8)	0.0369 (8)	0.0159 (6)	−0.0002 (6)	0.0053 (6)
C9	0.0415 (8)	0.0445 (9)	0.0379 (8)	0.0191 (7)	−0.0009 (6)	0.0030 (6)
C10	0.0509 (9)	0.0579 (10)	0.0426 (9)	0.0227 (8)	0.0099 (7)	0.0007 (7)
C11	0.0511 (9)	0.0554 (10)	0.0414 (8)	0.0257 (8)	0.0035 (7)	−0.0015 (7)
C12	0.159 (3)	0.0602 (14)	0.0657 (14)	0.0154 (15)	−0.0330 (15)	−0.0052 (11)
C13	0.153 (3)	0.0523 (14)	0.107 (2)	0.0143 (15)	−0.008 (2)	−0.0025 (13)
C14	0.0888 (16)	0.0643 (14)	0.0948 (17)	0.0370 (12)	0.0152 (13)	0.0253 (12)
C15	0.0470 (9)	0.0588 (10)	0.0342 (8)	0.0262 (8)	0.0012 (7)	−0.0008 (7)
N1	0.0539 (9)	0.0598 (9)	0.0764 (11)	0.0323 (8)	0.0161 (8)	0.0197 (8)
N2	0.0628 (10)	0.1065 (14)	0.0412 (8)	0.0480 (9)	−0.0084 (7)	−0.0060 (8)
N3	0.0474 (7)	0.0536 (8)	0.0364 (7)	0.0261 (6)	−0.0006 (5)	0.0010 (6)
O1	0.0840 (11)	0.1097 (13)	0.1024 (12)	0.0702 (10)	0.0338 (9)	0.0228 (9)
O2	0.0720 (10)	0.1099 (13)	0.0829 (11)	0.0555 (9)	−0.0105 (8)	0.0032 (9)
O3	0.0626 (8)	0.0875 (9)	0.0446 (6)	0.0495 (7)	−0.0111 (6)	−0.0084 (6)
O4	0.0791 (9)	0.0714 (9)	0.0649 (8)	0.0266 (7)	−0.0123 (7)	0.0114 (7)

### Geometric parameters (Å, º)

**Table:**

C1—C2	1.367 (2)	C9—N3	1.3354 (19)
C1—C6	1.396 (2)	C10—N3	1.4564 (19)
C1—H1	0.9300	C10—C11	1.475 (2)
C2—C3	1.376 (2)	C10—H10A	0.9700
C2—N1	1.472 (2)	C10—H10B	0.9700
C3—C4	1.377 (3)	C11—C12	1.315 (3)
C3—H3	0.9300	C11—O4	1.346 (2)
C4—C5	1.380 (2)	C12—C13	1.408 (4)
C4—H4	0.9300	C12—H12	0.9300
C5—C6	1.390 (2)	C13—C14	1.296 (4)
C5—H5	0.9300	C13—H13	0.9300
C6—C7	1.461 (2)	C14—O4	1.358 (3)
C7—C8	1.336 (2)	C14—H14	0.9300
C7—H7	0.9300	C15—N2	1.139 (2)
C8—C15	1.432 (2)	N1—O1	1.210 (2)
C8—C9	1.509 (2)	N1—O2	1.220 (2)
C9—O3	1.2170 (18)	N3—H3A	0.8600
			
C2—C1—C6	119.19 (15)	N3—C10—C11	114.03 (14)
C2—C1—H1	120.4	N3—C10—H10A	108.7
C6—C1—H1	120.4	C11—C10—H10A	108.7
C1—C2—C3	123.02 (15)	N3—C10—H10B	108.7
C1—C2—N1	117.60 (15)	C11—C10—H10B	108.7
C3—C2—N1	119.38 (15)	H10A—C10—H10B	107.6
C2—C3—C4	117.80 (15)	C12—C11—O4	109.03 (18)
C2—C3—H3	121.1	C12—C11—C10	132.98 (17)
C4—C3—H3	121.1	O4—C11—C10	117.98 (15)
C3—C4—C5	120.64 (16)	C11—C12—C13	107.3 (2)
C3—C4—H4	119.7	C11—C12—H12	126.4
C5—C4—H4	119.7	C13—C12—H12	126.4
C4—C5—C6	121.02 (15)	C14—C13—C12	106.8 (2)
C4—C5—H5	119.5	C14—C13—H13	126.6
C6—C5—H5	119.5	C12—C13—H13	126.6
C5—C6—C1	118.31 (14)	C13—C14—O4	109.9 (2)
C5—C6—C7	117.18 (13)	C13—C14—H14	125.0
C1—C6—C7	124.50 (13)	O4—C14—H14	125.0
C8—C7—C6	130.56 (13)	N2—C15—C8	177.71 (16)
C8—C7—H7	114.7	O1—N1—O2	123.45 (16)
C6—C7—H7	114.7	O1—N1—C2	118.39 (17)
C7—C8—C15	123.40 (13)	O2—N1—C2	118.15 (14)
C7—C8—C9	119.37 (13)	C9—N3—C10	122.71 (13)
C15—C8—C9	117.24 (12)	C9—N3—H3A	118.6
O3—C9—N3	123.55 (14)	C10—N3—H3A	118.6
O3—C9—C8	120.49 (13)	C11—O4—C14	106.99 (16)
N3—C9—C8	115.97 (12)		
			
C6—C1—C2—C3	0.6 (3)	C15—C8—C9—N3	−5.4 (2)
C6—C1—C2—N1	−179.20 (14)	N3—C10—C11—C12	−94.5 (3)
C1—C2—C3—C4	−1.1 (3)	N3—C10—C11—O4	84.68 (19)
N1—C2—C3—C4	178.69 (15)	O4—C11—C12—C13	0.6 (3)
C2—C3—C4—C5	0.5 (3)	C10—C11—C12—C13	179.9 (2)
C3—C4—C5—C6	0.5 (3)	C11—C12—C13—C14	−0.9 (4)
C4—C5—C6—C1	−1.0 (3)	C12—C13—C14—O4	0.9 (4)
C4—C5—C6—C7	178.99 (15)	C1—C2—N1—O1	−174.37 (16)
C2—C1—C6—C5	0.5 (2)	C3—C2—N1—O1	5.9 (2)
C2—C1—C6—C7	−179.50 (14)	C1—C2—N1—O2	6.8 (2)
C5—C6—C7—C8	177.38 (16)	C3—C2—N1—O2	−172.96 (17)
C1—C6—C7—C8	−2.6 (3)	O3—C9—N3—C10	−0.3 (3)
C6—C7—C8—C15	1.2 (3)	C8—C9—N3—C10	179.74 (14)
C6—C7—C8—C9	−179.14 (15)	C11—C10—N3—C9	−88.52 (19)
C7—C8—C9—O3	−5.1 (2)	C12—C11—O4—C14	−0.1 (3)
C15—C8—C9—O3	174.61 (15)	C10—C11—O4—C14	−179.50 (16)
C7—C8—C9—N3	174.88 (14)	C13—C14—O4—C11	−0.5 (3)

### Hydrogen-bond geometry (Å, º)

**Table:**

*D*—H···*A*	*D*—H	H···*A*	*D*···*A*	*D*—H···*A*
N3—H3*A*···N2^i^	0.86	2.27	3.056 (2)	152
C5—H5···O3^ii^	0.93	2.49	3.337 (2)	151
C7—H7···O3^ii^	0.93	2.49	3.362 (2)	156

Symmetry codes: (i) −*x*+1, −*y*+1, −*z*+2; (ii) −*x*, −*y*+1, −*z*+1.
