# Supplementary figures and images for: Crystal structure of 2-cyano-N-(furan-2-ylmeth­yl)-3-(3-nitro­phen­yl)propanamide
Source: Acta Crystallogr E Crystallogr Commun. 2015 Jul 15;71(Pt 8):o568–9. doi: 10.1107/S2056989015012918 (PMC4571401; doi:10.1107/S2056989015012918)

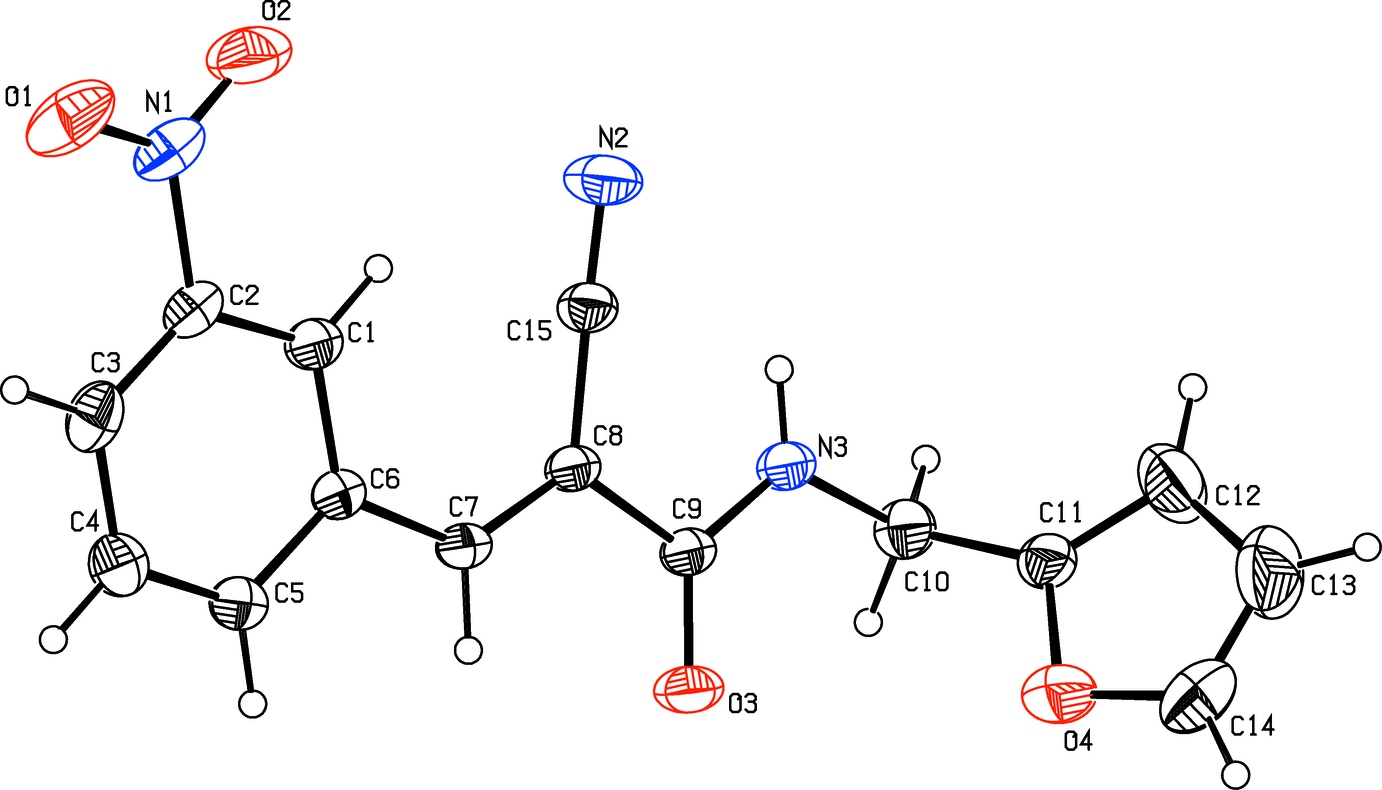

Supplement: Supplementary file 4 [file e-71-0o568-fig1.tif]

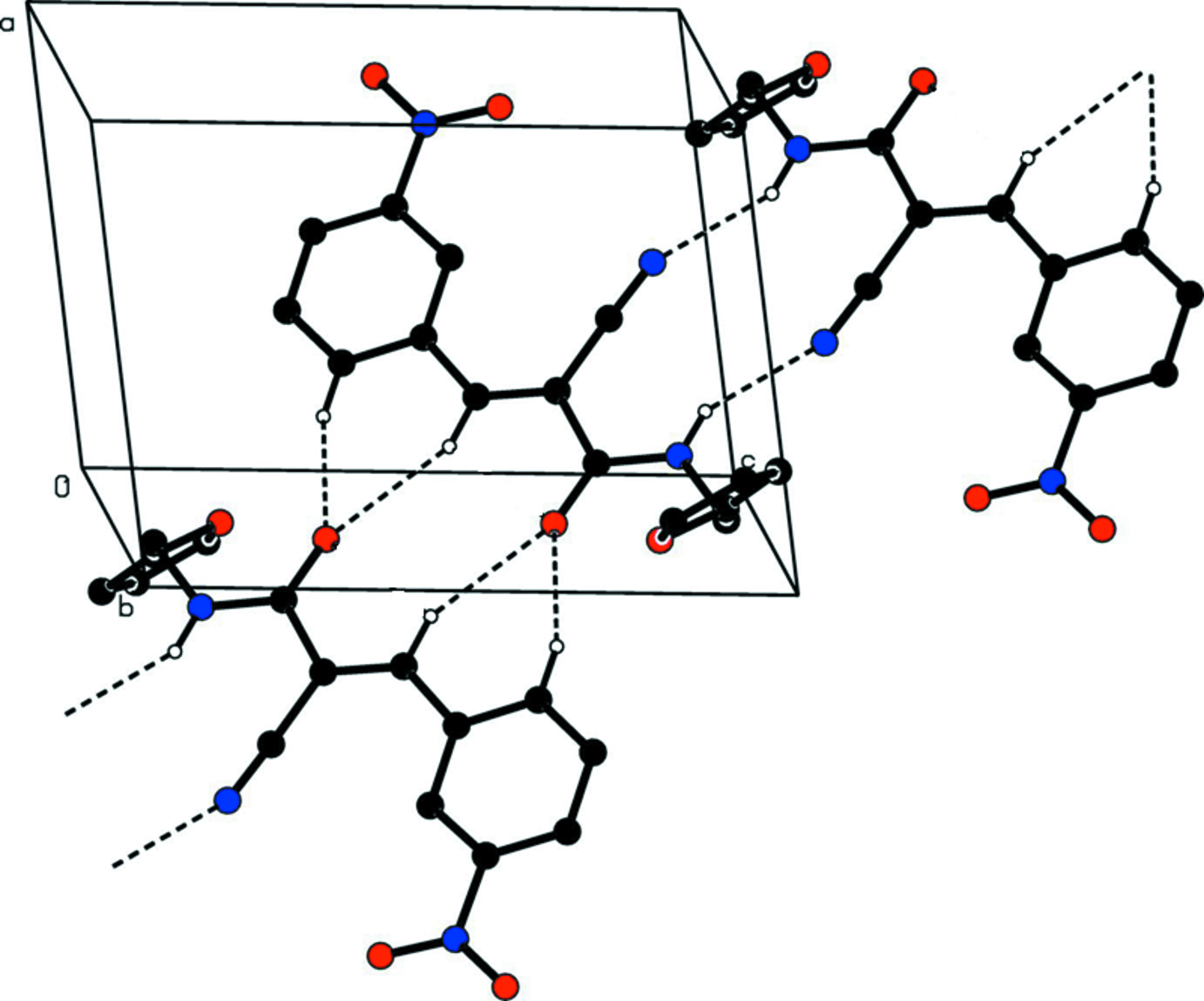

Supplement: Supplementary file 5 [file e-71-0o568-fig2.tif]
